# An Efficient and Secure Alert System for VANETs to Improve Crosswalks’ Security in Smart Cities

**DOI:** 10.3390/s20092473

**Published:** 2020-04-27

**Authors:** João Branquinho, Carlos Senna, André Zúquete

**Affiliations:** 1 Instituto de Telecomunicações, 3810-193 Aveiro, Portugal; joaobranquinho@ua.pt (J.B.); andre.zuquete@ua.pt (A.Z.); 2 University of Aveiro, 3810-193 Aveiro, Portugal; 3 DETI/IEETA, University of Aveiro, 3810-193 Aveiro, Portugal

**Keywords:** cryptography, secure communication, vehicular network, digital signatures, source authentication, hash chaining

## Abstract

A key characteristic of Smart Cities is the ability to reduce conflicts between different agents coexisting in a dynamic system, such as the interaction between vehicles and pedestrians. This paper presents a system to augment the awareness of vehicle drivers regarding the presence of pedestrians in nearby crosswalks. The proposed system interconnects Road Side Units (RSUs), which are informed about the state of the crosswalks, and vehicles, in order to spread to vehicles, the information about the presence of pedestrians in crosswalks. To prevent false information spreading, RSUs sign the alert messages they broadcast and all vehicles can validate the signatures. This poses strong security requirements, such as non-repudiation of alert messages, as well as strong real-time requirements, such as minimum message validation delays among vehicles approaching a crosswalk of interest. To manage the signed alert messages, we are proposing Nimble Asymmetric Cryptography (NAC), which authenticates implicit broadcast messages. NAC minimizes the usage of asymmetric ciphers, which are fundamental to assure non-repudiation but increase performance penalties and uses hash chaining for source authentication of implicit messages.

## 1. Introduction

Nowadays, road safety is a big concern among country governments. In Portugal, for example, in the year of 2017, there where a total of 34,416 traffic accidents. These accidents resulted in a total of 43,893 injured people and 602 deaths. Of those 34,416 accidents, 5651 involved pedestrians, 42.7% of which happened in signaled crosswalks. These statistics show that a significant part of road accidents happen on signaled pedestrian crosswalks.

Although there are some studies on how to provide safety between pedestrians and vehicles in crosswalks through Vehicular Ad-hoc Networks (VANETs) [1,2,3,4,5], there are important challenge relationships with authentication and privacy. Moreover, in VANET environments, with intermittent network connections, reduced contact times, and quick topology alteration, the opportunities for attack are numerous.

In order to deal with these challenges, we are proposing a solution with a focus in two aspects: to inform vehicles about the presence of pedestrians in a crosswalk in a secure fashion and to assure that vehicles can validate that information using a very efficient method. For reaching this goal, we assumed that crosswalks have a trustworthy pedestrian detection system [6], which they use to gather information to convey to a nearby Road Side Unit (RSU). This information is then spread along a variety of vehicles of interest in the neighborhood of the source crosswalk using the VANET formed by the vehicles themselves.

To prevent false information spreading, alert messages broadcast by RSUs must be authenticated, and all vehicles (or their On-Board Units (OBUs)) must validate them. This poses strong security requirements, such as non-repudiation of alert messages, as well as strong real-time requirements, such as minimum message propagation delays among vehicles approaching a crosswalk of interest.

Considering that RSUs and OBUs must perform several computationally intensive parallel tasks, such as

(i) Maintaining an Internet connection,
(ii) Control of car sensors and,
(iii) Driver assistance mechanisms.

Our solution uses hash chaining for origin authentication in order to consume less resources. Despite this, we still use asymmetric ciphers, which are fundamental to guarantee non-repudiation, but we minimize its usage to significantly decrease performance penalties. Both these features were combined in a novel protocol, the Nimble Asymmetric Cryptography (NAC), which was designed to efficiently and timely authenticate implicit broadcast messages [7], used to inform if there are pedestrians around the crosswalks or not.

This paper provides an extended overview of solutions designed to authenticate messages in VANETs (Section 2), in order to show why NAC is a valuable solution to tackle the authentication of crosswalks’ state information broadcast to vehicles (Section 3). Then, we provide a detailed evaluation of the exploitation of NAC in a simulation performed over a real crosswalk scenario (Section 4). This simulation allowed us to evaluate several deployment details, such as the impact of the number and location of RSUs handling a set of existing crosswalks (Section 5). Furthermore, it allowed us to study NAC enhancements, such as the aggregation of multiple implicit messages, and to study the impact of the hash chains’ depth used in implicit messages. Finally, Section 6 shows the conclusion and future directions of the work.

## 2. Related Work

According to ETSI TC ITS [8,9], the security in VANETs must consider authentication, integrity, non-repudiation, privacy, confidentiality, authorization, real-time constraints and availability. Scenarios with VANETs and IoT must take into account special requirements such as low computational overhead and low communication overhead.

For instance, in [10,11,12,13,14,15,16], the authors propose Group Signature (GS) schemes for signing messages in VANET scenarios, where any member of a group signs a safety message with its private key, while the receiver of the message uses the group public key to validate the authenticity of the message. Although important properties like scalability, unlinkability and unforgeability are achieved, the usage of group signatures leads to computational overhead, and consequently, it can lead to message losses.

Some solutions are based on shared-key cryptography, where a single, secret key is shared between the sender and the receiver. The authentication schemes using shared keys can explore them using three different approaches [3]: Message Authentication Code (MAC), Hash Functions and TESLA schemes.

In cryptography, a MAC is a short piece of information used to authenticate a message given a shared, secret key. MAC algorithms are particularly useful as they allow key owners to confirm both data integrity and data authenticity. However, they do not provide for non-repudiation, given that any of the key holders can repudiate the generation of a particular MAC computed with the key they own. In [17], Lin et al. introduced an authentication scheme based on MACs that present privacy preservation properties. This scheme combines the use of a MAC algorithm and a hash chain element to calculate MAC tags. It reduces the computational overhead by minimizing the usage of asymmetric message signing and validation. This scheme also minimized bandwidth usage when compared with other asymmetric signature schemes, given that MACs are usually smaller than signatures produced with asymmetric cryptography. In [18], Zhang et al. proposed an authentication scheme known as Roadside Unit Aided Message Authentication Scheme (RAISE). This scheme establishes a shared key between each vehicle and the nearest RSU. Whenever the sender vehicle wants to send a safety message, it broadcasts it to other vehicles. The RSU is responsible for verifying the authenticity of the message and communicates the result to the vehicles. By introducing a MACs, RAISE aims to reduce both communication and communication overheads. While lightweight approaches, the above solutions have no particular concern with the possibility of losing important messages.

There are works that use hash function authentication schemes with the expectation of obtaining more efficient execution times. However, this scheme does not provide full authentication nor non-repudiation, given that an attacker may edit the message and update the hash value, and produce a completely new message. In [19], Chuang et al. introduced Trust-Extended Authentication Mechanism (TEAM), a decentralized alternative that uses a pre-shared key in the authentication process. In this scheme, before a vehicle can join a VANET, its OBU must register with an authentication server to perform a key agreement. When a vehicle wants to access a service, it has to perform the login procedure, check the authentication state itself, and if the lifetime of the key is reduced to zero, the vehicle is considered mistrustful. The trustful vehicles assist other vehicles in performing the authentication procedure, in communicating with other trustful vehicles or in accessing the Internet. If the key is below a given threshold, the vehicle performs a key update procedure. TEAM minimizes the usage of asymmetric keys in order to establish a single multi-usage symmetric key. In [20,21], researchers presented schemes that make use of hash functions to achieve special security requirements, such as privacy, unlinkability and untraceability. Vighnesh et al. [21] presented an authentication process that takes place at a trusted third party site (Authentication Centre—AC). Each safety message is attached with an authentication code by the sending vehicle. The receiving vehicle decrypts an authentication code by a pre-loaded public key of an AC. The use of a third party in authentication potentially increases response time and may not meet the time requirement of VANETs. Hash function schemes provide a lightweight solution that fits under the VANET particular needs. Nonetheless, most of the existing schemes have no concern for lost messages, a common scenario in vehicular networks, and therefore, they are not suitable for emergency message scenarios.

A topic to be considered in security schemes for VANETs is the low computational capacity of the nodes (OBUs and RSUs). In this regard, it is important to analyze security works targeting IoT environments [22,23]. We also considered works related to our case use, safety in pedestrian road crossing in smart cities. Dim et al. [24] proposed a street-centric protocol that explored the effect of traffic lights on vehicle distribution in a street to calculate street connectivity based on the distribution and density of vehicles in the middle area of a street. Xia et al. [25] proposed a greedy traffic light and queue aware routing protocol (GTLQR), which jointly considers the street connectivity, channel quality, relative distance, and queuing delay to alleviate the packet loss caused by vehicle clustering at the intersection and balance the traffic load among vehicles. Some works, such as [26,27], deal with the detection of pedestrians in crosswalks as an improvement to smart cities, yet they focus on the application itself and do not include security measures related to information transmission.

Some solutions use a hybrid approach combining several techniques. Yang et al. [22] proposed a lightweight privacy-preserving authentication scheme to enhance the communication security in VANETs, which takes advantage of the Modular Square Root (MSR) technique. MSR technique is a public key cryptography system based on the difficulty of factorization similar to Rivest, Adleman and Shamir (RSA). One of the prominent advantages of MSR is the much simpler encrypting operation (modular squaring) than RSA and Elliptic Curve Cryptography (ECC). Kang et al. [28] proposed forward an access control with an authentication scheme for disseminated messages in VANETs. The scheme integrates a pseudonym with an identity based signature that could not only authenticate the messages in vehicular communication but also protect the privacy of message generators. They apply batch verification and adopt ciphertext policy attribute-based encryption to provide an access control service to set expressive and flexible access structure for the specified vehicles in VANETs communication. However, the proposed scheme does not describe communication costs and verification delay, and also does not provide traceability in case of dispute.

The Guy Fawkes protocol [29] by Anderson et al. is a secure communication algorithm based on hash functions and message chaining mechanisms. The protocol works by chaining messages, i.e., including the hash of a nonce in the *i*-th message and in message i+1 revealing that nonce. The Guy Fawkes protocol is straightforward, yet the chain alone does not provide enough security. The non-repudiation criterion is only accomplished if the first message source can be proved to be from the supposed sender. Without this constraint, the receiver has no certainty about the source of the message, and therefore, the whole chain cannot be trusted. With this in mind, the bootstrap of the protocol must use a digital signature protocol that authenticates the message chain source. Only the first message requires signing so that the whole chain is trustworthy. We commonly refer to this message as an anchor message. Another issue of this protocol presents itself when we consider its usage in unreliable networks. However, the unpredictability of unreliable networks poses a severe challenge to the Guy Fawkes protocol since if a single package is lost, the whole chain must be reset. A way to work around this is to send an anchor message periodically, so nodes that lost messages and new nodes would be able to reestablish the message chain. Moreover, and although this protocol presents security features that can lead to authenticated communications, under specific circumstances, it is vulnerable to man-in-the-middle (MitM) attacks. Clock synchronization and fixed reception windows can avoid this problem, but they impose a fixed transmission rate that increases the medium occupancy.

In [30,31], Perrig et al. presented Timed Efficient Stream Loss-tolerant Authentication (TESLA), a protocol that proposes an authentic message broadcast system using symmetric cryptography. This solution identifies the problem with message tampering associated with the Guy Fawkes protocol. TESLA uses time (delayed key disclosure) to achieve the asymmetry property required for secure broadcast authentication. TESLA uses hash chains and different time slots of the available time. In a hash chain, each item acts as a synchronous key, and each key is used only once per time slot for MAC computation at the sender. These computed MACs are disclosed to the recipients in the next time slot through broadcast [3]. The main features of TESLA are: low sender and receiver computation overhead (around one MAC function computation per packet), low communication overhead, and perfect robustness to packet loss. TESLA requires loosely synchronized clocks between the sender and the receiver, and the authentication is slightly delayed [32]. Although the usage of TESLA avoids message loss problems, Perrig et al. predicted some Denial of Service (DoS) involving the Buffering of messages on the receiver side. TESLA is vulnerable to memory-based Denial of Service attacks. In TESLA, receivers store data until the corresponding key is disclosed. Malicious parties can flood receivers with invalid messages that will never have a corresponding key disclosure. This poses as a pollution attack to the receiver. Jahanian et al. [33] utilizes a timed model checking approach based on timed colored Petri nets to model and verify TESLA considering its time-sensitive behaviors. The proposed solution shows how neglecting timing aspects in protocol design and in protocol modeling can lead to successfully launched attacks and erroneous analyses, respectively, and how its refinement can help improve the protocol’s security. The TESLA++ protocol by Perrig et al. is an improvement to the TESLA protocol that addresses memory pollution attacks. In TESLA++, the receiver self-generates the MAC, which reduces memory requirements. Just like in TESLA, this requires that the nodes are loosely time-synchronized. It is not a problem in WAVE networks, given that these networks implement time synchronization. Although the broadcaster buffers messages, it is important to notice that messages are validated ***d*** time stamps after their disclosure. TESLA++ improves TESLA by preventing DoS attacks, yet messages are validated with delay. This is one of the reasons that compelled us to create our solution: Nimble Asymmetric Cryptography (NAC).

## 3. Nimble Asymmetric Cryptography Protocol (NAC)

Taking into account the characteristics of VANETs (mobility, intermittent connectivity, long and variable delay in information delivery), it is important that message validations are performed even if a node loses one or more packets. Furthermore, message losses also impose a variable, and potentially high, delay when validating incoming messages when relying on centralized Trusted Authorities, as in [4]. In scenarios where delay is critical, such as in alarm delivery when lives are at stake, the time to validate a message must necessarily be predictable and bound.

The Guy Fawkes, TESLA, and TESLA++ (see Section 2) can improve the efficiency of message authentication by using symmetric keys to perform integrity validations. Nevertheless, they are not suitable to be used in our crosswalk scenario due to their delayed message validation approach. This motivated the design of Nimble Asymmetric Cryptography (NAC), a protocol inspired by the Guy Fawkes protocol, but that does not impose message validation delays.

Some IoT sensors can naturally produce a sampled value belonging to a reduced set of possible alternatives. For instance, traffic lights can be green, yellow or red, or even turned off, but we usually do not have other states. Thus, two bits were enough to code the state of a traffic light. The same happens with crosswalks since they can transmit a message that ultimately can be reduced to two cases: either someone is using the crosswalk or no one is there. Thus, a single bit would be enough to code a message from a crosswalk. NAC was designed in accordance with this characteristic: an implicit message belonging to a set of cases is provided by a value belonging to a chain of values bound to such an implicit message.

A preliminary version of NAC was previously published in [7]. The description below includes new features that were added to the original design.

### 3.1. Protocol Overview

We assume that we have a network with two types of actors:

(i) One sender (the information origin) and
(ii) Multiple receivers (the ones that will benefit from the information).

Over time, the sender broadcasts two types of messages: anchor messages and implicit messages. These messages convey different kinds of information but always from a limited set of possibilities. Due to its broadcast nature, there are no reception acknowledges. The communication channel is purely unidirectional; thus, losses are not perceived by senders.

**Figure 1:**
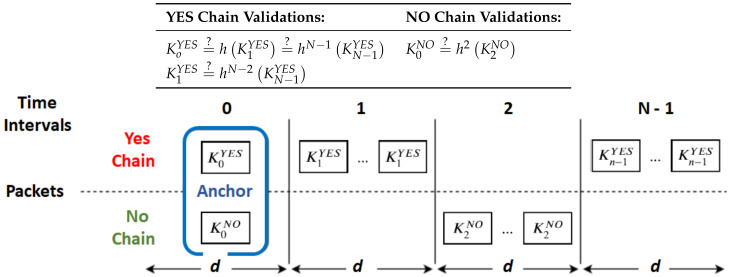
The Nimble Asymmetric Cryptography (NAC) protocol execution pattern. The keys $K_{i}^{msg}$, where *msg* is either YES or NOT, are computed as follows: $K_{i}^{msg}=h^{\delta }\left(K_{i+\delta }^{msg}\right)$.

NAC uses reverse hash chains to achieve a fast message authentication (Figure 1). Most broadcast messages are implicit messages, which contain values of those hash chains. These chains are bound to fixed messages, and that is why their values provide an implicit message. Hash chains end on signed values ($K_{0}$ values in Figure 1) transmitted from time to time in bigger, anchor messages. These are the ones that allow receivers to ultimately validate implicit messages, i.e., to check if they are correct and come from an authenticated source.

### 3.2. Anchor Messages

Anchor messages provide the data required for receivers to interpret and validate subsequent implicit messages. Namely, they carry the identity of the information source (e.g., a crosswalk identifier), the $K_{0}$ value of each hash chain associated to each given message from that source (e.g., YES and NO in Figure 1), the validity period *d* for each implicit message and the signature of the sender. Furthermore, they also state which of the chain values is the actual message for its interval (YES or NO for time interval 0 in Figure 1).

Anchor messages must ensure non-repudiation properties for themselves and transitivity to the chain of implicit messages following them. The widely used methods to achieve these properties are digital signatures and public-key certificates. In this work, we assume that recipient nodes have the public key of the sender.

### 3.3. Implicit Messages

Implicit messages are fundamentally hash chain values and the identity of the information source; the hash chain they belong to defines their message. For instance, relative to Figure 1, $K_{i}^{NO}$ give a NO message for time interval *i*.

During a given time interval (with a duration *d*), a source can only transmit one kind of implicit message. It can transmit it several times, to cope with transmission losses, but is always the same. Critical messages (e.g., occupied crosswalk) can be transmitted more often than non-critical ones (e.g., empty crosswalk).

The usage of implicit messages has the advantage of transmitting less data (the size of a hash is smaller than most of the signatures produced by digital signature protocols) and cryptographic hash functions are usually extremely fast and faster than signature validations (namely, because that also requires a hashing operation).

The value of *d* and the repetition of implicit messages are implementation parameters. Implementations may also include indexes in implicit messages to accelerate their validation (for example, to immediately discard messages received too late, beyond their internal time). As we will see further ahead, implicit messages do not need to refer to the chain they belong to because that will not accelerate the validation process and increases the message size.

### 3.4. Validation of Implicit Messages

**Figure 2:**
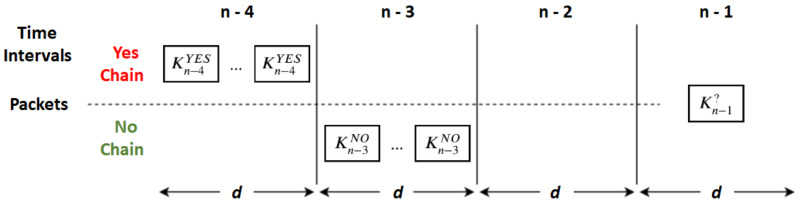
Recipient validation process upon receiving a new implicit message ($K_{n-1}^{?}$) given a past of validated and accepted messages ($K_{n-4}^{YES}$ and $K_{n-3}^{NO}$).

In Figure 2, we depict the validation process on the recipient side for an implicit message received in interval n−1, given that:

- In interval n−4, it received implicit messages belonging to the *YES* hash chain ($K_{n-4}^{YES}$);
- In interval n−3, it received implicit messages belonging to the *NO* hash chain ($K_{n-3}^{NO}$); and
- In interval n−2, for some unknown reason, the recipient received nothing.

In order to validate the received message, the recipient needs to hash it at least two times. If $K_{n-3}^{NO}=h^{2}\left(K_{n-1}^{?}\right)$, then it is a *NO*. Otherwise, it hashes it once again and tests if $K_{n-4}^{YES}=h^{1}\left(h^{2}\left(K_{n-1}^{?}\right)\right)$. If the equality test fails, the message is invalid and is discarded; otherwise, it is accepted as a *YES* message and stored to validate subsequent ones. For each chain from a given source, the recipient only needs to keep the last validated implicit message for validating new ones. Thus, for a source providing *D* different messages, only *D* implicit messages need to be kept for performing future validations.

**Figure 3:**
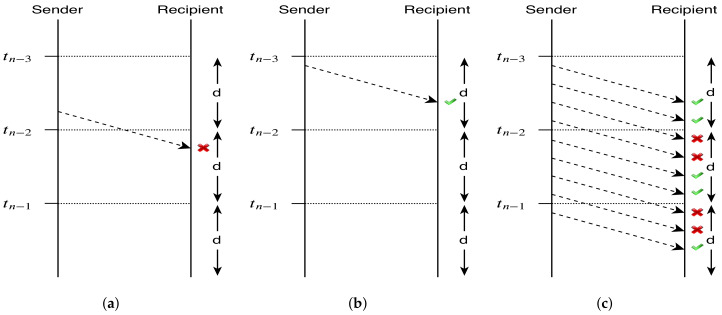
Time restrictions to implicit message validity. (**a**) Invalid implicit message. (**b**) Invalid and valid implicit messages. (**c**) Invalid and valid implicit messages overtime.

For a message that was sent in validity interval *i*, to be considered valid, it must be received in the same validity interval *i*. Only this way, the recipient is sure that the received message has been sent by the supposed sender (the only one who knows $h\left(K_{i+1}\right)=K_{i}$ at that instant). In Figure 3, we depict time restrictions to the validity of implicit messages.

### 3.5. Hash Chain Lengths and Intermediate Anchor Messages

The maximum length of hash chains defines the minimal frequency to send anchor messages. In other words, when a new chain of *N* values is created by the sender (by hashing from a random $K_{N-1}$ until $K_{0}$), the sender produces an anchor message with $K_{0}$, sends it, and then can use implicit messages to convey information to receivers during $\left(N-1\right)\times d$ time. After that, it needs to send a new anchor message with new chains.

The overhead added by asymmetric cryptography required to handle anchor messages is redeemed by implicit messages. This means that longer chains are more effective in reducing the cryptographic overheads of NAC. The same also happens for data transmission overheads, since anchor messages are bigger than implicit messages. However, longer hash chains may increase the cost of validating hash chains under heavy packet losses as well, and it also increases the issues related to the loss of anchor messages. In fact, anchor messages are fundamental to enable receivers to trust implicit messages because it allows them to follow hash chains until reaching a signed value (or other chain values previously validated the same way, such as $K_{1}^{YES}$ or $K_{2}^{NO}$ in Figure 1). Therefore, if a receiver loses an anchor message, it cannot trust the following implicit messages. This can be an issue for critical messages since receivers may be in the dark for $\left(N-1\right)\times d$ time upon losing an anchor message.

**Figure 4:**
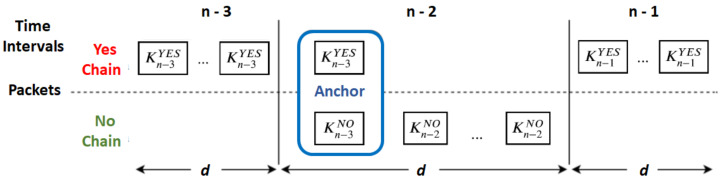
Example of an intermediate anchor message transmitted for time interval *n* − 2.

To tackle this problem, implicit messages can be arbitrarily replaced by intermediate anchor messages (Figure 4). These are equal to ordinary anchor messages but are generated for intermediate values of hash chains. For instance, an intermediate anchor message transmitted for the time interval *T* will replace a $K_{T}$ implicit message and will contain all the $K_{T}$ values for the same source. Intermediate anchor messages allow receivers that lost the initial anchor message to get synchronized with subsequent hash chains some time in the future but before changing to new chains. This way, we can reduce outage periods upon the loss of anchor messages, while using long chains.

What is, then, the advantage of using intermediate anchor messages instead of shorter hash chains? The main advantage is that we can provide an alternative anchor message to the recipients that lost the initial anchor message without adding any validation overhead to the other recipients.

In fact, for the receivers that got a previous anchor for the same chains, either the first one or some other intermediate, they can check the actual message that an intermediate anchor conveys without validating its signature; they only need to use some previously received implicit messages.

On the other hand, for those that had not received any anchor for the current chains, intermediate anchors allow them to start validating those chains. This way, we can increase the number of actual receivers without re-transmitting the initial anchor (for loss tolerance) and without increasing the validation time for those that are already validating the chains.

Thus, by using hash chains with length *N* and *A* anchor messages (including initial an intermediate), we have roughly the same loss tolerance that we would have *A* independent chains with length $\frac{N}{A}$ but with savings on signature validations on the receivers that got a previous anchor message for the same chains. On the other hand, the number of extra anchor messages demands more bandwidth and more computational power on the sender side, and that is the reason for using implicit messages most of the time.

Note, however, one subtle detail. If a recipient gets an intermediate anchor with a message that can be validated against the previously received implicit messages (e.g., with a $K_{i}^{YES}$ that can be validated against a previously received $K_{i-1}^{YES}$), its other intermediate chain values cannot be all trusted just because one of them was validated against a previous one (i.e., $K_{i}^{NO}$ cannot be trusted because $K_{i}^{YES}$ was validated). In fact, that would allow an attacker to create a false intermediate anchor from an implicit message, for the same interval, in order to benefit from that wrong trust assumption (i.e., the attacker could fabricate a fake intermediate anchor with $K_{i}^{YES}$ and a fake ${K^{\prime }}_{i}^{NO}$). To trust on all intermediate chain values provided in an intermediate anchor, the recipient needs to hash them all until reaching a trusted one, or validate the anchor’s signature.

### 3.6. Clock Synchronization

NAC requires clock synchronization between senders and receivers in order to validate anchor messages, but not implicit messages. Otherwise, attackers could create chaotic situations by re-transmitting previous anchors and related implicit messages. To this end, anchor messages must carry a timestamp that is checked against the local clock as a first validation step.

Fortunately, most Wireless Access in Vehicular Environments (WAVE) communication protocols already contemplate time synchronization. IEEE Standard for Wireless Access in Vehicular Environments 1609 [34] is an example.

## 4. Proposed Use Case and Test Platform

The NAC protocol poses as an attractive approach to our crosswalk scenario. As referred earlier, a great deal of traffic accidents occurs in crosswalks. Every aspect of the crosswalk must consider efficient and lightweight alternatives to the traditional solutions used in authentication scenarios. The NAC protocol also takes into account the mobility, contact intermittency between nodes and constant topology change of networks present in VANETs environments. The main characteristic of the protocol that makes it a good option in VANETs is the use of hash chains and the fault tolerance provided to temporary connectivity issues.

NAC also minimizes the exploitation of asymmetric cryptography. This minimization occurs on the sender side, when signing, and on the recipient side, when validating the signature. The signing/validating process, when using our protocol, is substituted by a sequence of executions of a hash function. Hash functions are used with the intent of providing a high-speed message validation, since asymmetric cryptographic algorithms are known to be relatively slow. Later on, we will specify which cryptographic technologies we will use in the signature process and depict exact timing gains when comparing anchor message validations and implicit message validations.

Another significant factor is the messages’ size. While anchor messages are able to carry signed information, they are bigger than implicit messages. Later on, we will provide a more detailed study on this matter.

**Figure 5:**
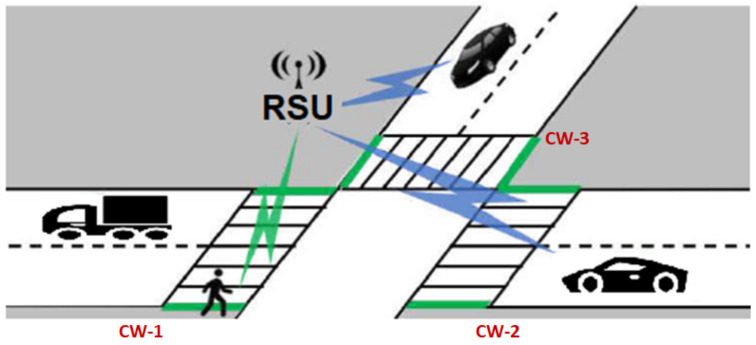
Crosswalk scenario.

In the crosswalk scenario (Figure 5), the senders are the RSUs and recipients are OBUs (vehicles). With this in mind, we can consider that messages relative to several crosswalks can be packed in a single one. This becomes an interesting factor when wireless protocols are subject to collision control of their channels. This way, packed messages contribute to a less noisy wireless environment for the VANET. Moreover, this allows RSUs to regulate the signal strength so that messages only reach relevant vehicles.

### 4.1. Message Content

The NAC protocol broadcasts two types of messages: the anchor messages asymmetrically signed and the implicit messages, which are symmetrically signed through the use of hashes. Anchor messages are asymmetrically signed and, therefore, can carry more information regarding both the crosswalks and RSUs. Symmetric messages are only able to carry information regarding the current state of the crosswalk.

Anchor Messages contain relevant information about the RSU, each crosswalk and timing details on protocol’s settings. While anchor messages are significantly larger than implicit messages, the number of broadcast anchor messages is reduced compared with implicit messages. For each crosswalk inside your coverage area, the RSU sets the following in each anchor message per crosswalk:

- id—The identifier of the crosswalk;
- Current state—The current state of the crosswalk and can either be *occupied* or *not occupied*;
- Keys—The hash chain values relative to the *occupied* and *not occupied* states of the crosswalk;
- CW Latitude—The crosswalk latitude; and
- CW Longitude—The crosswalk longitude.

The RSUs also adds the following information about itself and the subsequent transmission of messages:

- RSU id—The identifier of the transmitting RSU;
- Unique id—A incremental unique identifier for that message;
- Current timestamp—The timestamp of the message transmission instant, in milliseconds;
- fValidity interval size—Validity interval, in milliseconds (*d*);
- Current interval—Index of the current validity interval;
- Base time—Time stamp of the next validity interval;
- Longitude—The longitude of the RSU.

Anchor messages also carry the digital signature over all the above parameters. Geographic coordinates may be used by the vehicle to calculate distances and predict which of the crosswalks may be relevant. This information was included in anchor messages, but it could be fetched in advance for a given area of interest.

Implicit messages are much smaller when compared to anchor messages. They carry information about one or more crosswalk, and for each it contains:

- id—The crosswalk identifier; and
- key—The hash chain value that yields the occupancy information.

Moreover, this message also carries the identifier of the RSU that is disseminating the information. Notice that by including the identifiers of the RSU and the crosswalks in the message, NAC allows vehicles to decide which crosswalks they must confirm.

### 4.2. Radio Medium Occupancy

The bandwidth occupancy was one of the concerns behind the NAC exploitation for transmitting crosswalk information to vehicles through a VANET. Considering that each RSU could disseminate information regarding the state of several crosswalks at the same time, we explored NAC in a way that allows us to aggregate multiple messages, from different sources, in each single message (either anchor or implicit). This aggregation optimizes the use of RSUs by minimizing installation costs and decreases network contention. This way, it also promotes the scalability of the entire system. Moreover, crosswalks are usually located at junctions and roundabouts, composing distinct geographically separated clusters. This reality facilitates RSU positioning to reach a significant number of vehicles of interest for each crosswalks’ cluster.

RSUs may distribute information to nearby OBUs in a way that minimizes the number of OBUs for which the received information is useless. For this end, an RSU can:

(i) Limit the radio signal power to reduce the transmission range (because for vehicles to far away from the crosswalks at stake it would be useless); and
(ii) Use directed and non-directed broadcast transmissions to focus the information transmission only on the vehicles of interest.

This way, the system is able to minimize the number of useless message validations and minimize its overall impact in the VANET communications.

### 4.3. On-Board Information Processing

RSUs use NAC to securely broadcast the current state of a set of supervised crosswalks. It is up to the receiving vehicles the responsibility to decide if that information will (or not) be useful to them in a near/distant future. This decision may use several methods, such as the current GPS location of the vehicle and the vehicle’s route.

### 4.4. Message Dissemination

Many emergency message protocols, such as [35], use message dissemination strategies to guarantee emergency message dissemination over the VANET network. However, applied to our crosswalk scenario, we consider that message re-transmission by the OBUs should be disregarded. We advocate this behavior based on reaction and braking distances and the perimeter where the information is useful (relevance zone).

Equation (1a) allows us to calculate the total stopping distance of a vehicle based on its velocity (*v*) [36].

(1a) $$d_{total}=d_{braking\_dist}+d_{react\_dist}$$

(1b) $$d_{react\_dist}=v*t_{reac}$$

(1c) $$d_{braking\_dist}=\frac{v^{2}}{2\mu g}$$

The reaction distance, product of vehicle velocity (*v*) and reaction time ($t_{reac}$)—Equation (1b), is the distance it takes between realizing the danger presence and starting to break the vehicle. The braking distance starts when the driver starts braking until the vehicle fully stops. The braking distance depends on vehicle velocity (*v*), and friction coefficient (μ), as given by Equation (1c).

**Figure 6:**
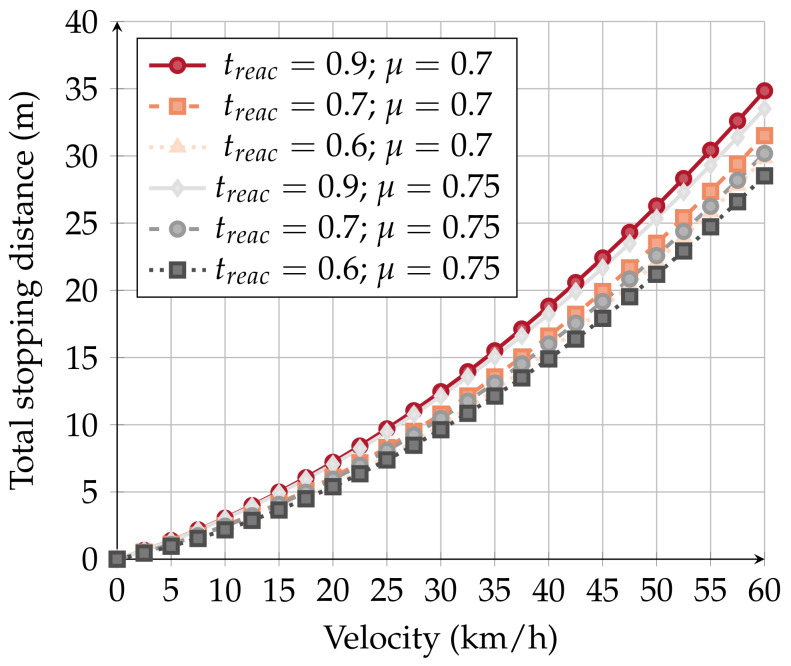
Braking distances.

Equations (1b) and (1c) contain several variables about which we can make assumptions. Under normal driving conditions, the reaction time is considered to be 0.7 [36]. The friction coefficient may vary with the quality of the vehicle, weather conditions and the driver. For testing purposes, we consider a friction coefficient of 0.75 [37]. Figure 6 illustrates the possible braking distances considering different velocities. Moreover, we also considered other friction coefficients and reaction times, which, according to [36,37], are also valid.

Different situations may have multiple impacts on the braking system. Factors like meteorology, the age/state of mind of the driver, and the quality of the car impact of the total stopping distance. Considering the several possible variations, at 60 km/h, a vehicle can stop 35 m after being warned (in a pessimist case).

In our application scenario (Section 5.3), the RSU can reach up to 100 m in an urban situation. Considering that an inattentive driver can take up to 35 m to stop the vehicle that an RSU can reach up to 100 m, we may safely conclude that there is no need for message dissemination strategies. Cars are warned in time about the state of the crosswalk, and NAC message re-transmission by the OBUs would only contribute to radio environment pollution.

### 4.5. Test Platform

**Figure 7:**
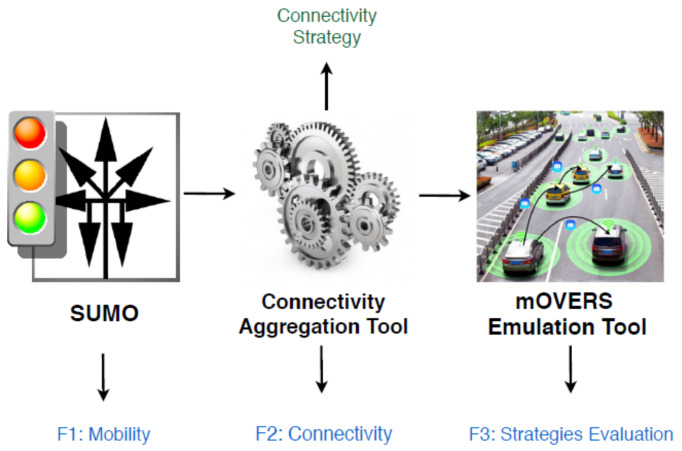
Software testbed platform.

The test platform consists of three tools: Simulation of Urban MObility (SUMO), Connectivity Aggregation Tool (CAT) and mobile Opportunistic Vehicular Emulator for Real Scenarios (mOVERS) (Figure 7).

Since the strategies for exploring NAC for disseminating the state of crosswalks to the vehicles of interest need to be tested in a dense and controlled scenario, we opted to perform these tests in the mOVERS emulator [38]. In this emulator, we can provide the vehicles’ mobility and the pedestrians’ mobility at nearby crosswalks in a given scenario. mOVERS was developed to emulate a VANET using real mobility and connectivity data from the Vehicular Network of Porto, BusNet [39]. The code executed in each OBU and RSU in the real vehicular network is the same that is executed in the emulator.

While mOVERS was designed to work with real mobility data collected from the Porto network, we could not use such data for evaluating NAC for several reasons. One of them is that it does not contain anything about pedestrians, and the second is that it only contains mobility data relative to the VANET’s OBUs, of which there are not many, and not any other vehicle.

Consequently, we chose a different scenario (in the Aveiro city) with an interesting set of crosswalks, we generated the mobility of vehicles with Simulation of Urban MObility (SUMO) (http://sumo.dlr.de/index) and the mobility of pedestrians with a dedicated SUMO module (https://sumo.dlr.de/docs/Simulation/Pedestrians.html), we established the connectivity of those vehicles (their OBU) with RSUs with the Connectivity Aggregation Tool (CAT) and the resulting data was provided to mOVERS in order to evaluate the performance of NAC.

CAT creates connectivity data between OBU and RSUs based on their distance: when closer than a threshold distance, they are connected, otherwise, they are disconnected. For our case, we considered 100 m for that threshold, which is about three times higher than the minimum warning distance of 35 m previously established. This means that the power of RSU transmissions of NAC messages only needs to be high enough to reach 100 m with a small probability of message losses; more than that is useless and even negative, as it increases collisions and unwanted media occupancy.

The connectivity data produced by CAT establishes that a given node is reachable by another one in a given time frame. However, the way mOVERS work is different: it requires each node to have a time-variable table of neighbors with their actual Received Signal Strength Indicator (RSSI). Since mOVERS only uses these RSSI values on a Boolean fashion (with connection when above 15 dBm, without connection otherwise), CAT creates entries on nodes’ neighbor tables with an RSSI value above 15 dBm when nodes are closer than the threshold distance (100 m), and does not create entries otherwise.

The CAT tool also establishes the relation between each crosswalk and an existing RSUs. In real scenarios, it is common for RSUs to be responsible for multiple information provider devices. Usually, they carry adequate computational capability in order to do so. Taking this into account, we assigned to each crosswalk the nearest RSU. This RSU will be responsible for providing the OBUs with authenticated information about the state of the crosswalks it is responsible for.

mOVERS was developed in C++ and designed to facilitate the integration of new modules in order to be extensible. Moreover, it supports several communication protocols such as WAVE, Wireless Fidelity (Wi-Fi) and IEEE 802.11a/b/g. We customized mOVERS for our scenarios.

For vehicles to be informed in real-time about the state of the crosswalks, it is essential that validity intervals are small and, consequently, message disclosure intervals even smaller. With this in mind, we adapted mOVERS to allow for millisecond time variations. In our close-range messaging scenarios (relevance zone of 100 m), vehicles near the crosswalks must receive messages without delays. To this simplistic scenario, we modified the mOVERS’ routing protocol to broadcast in a single-hop (i.e., NAC messages are never re-transmitted by receivers). The RSUs are responsible for disseminating messages only to their neighbor vehicles (OBUs). The OBUs interpret and decipher the messages, identifying the existence of crosswalk pedestrians.

SUMO has a module that allows the generation of pedestrian mobility and integration with vehicle scenarios. However, we gave up on this solution for a simple reason: the inclusion of pedestrians in our mobility scenario would have a direct impact on vehicle mobility. By including pedestrians in the mobility scenarios, upon spotting them, the vehicles instantly reduce their velocity. This has a cascade effect on subsequent vehicle’s geographic coordinates. Bear in mind that our work aims to provide crosswalk warnings to inattentive drivers. By including pedestrians in the SUMO simulation, we instantly make the drivers aware of the existence of pedestrians. Taking this into account, we added the pedestrian mobility in the mOVERS platform. Along with the mobility data, we provide SUMO with crosswalk data, including identification, geographic positioning and length. After this, we calculate the time a pedestrian remains in the crosswalk (based on a mean velocity from [40]) and a random time in which the crosswalk has no pedestrians. This way, we emulate the pedestrian presence that is broadcast by the RSUs for their neighboring OBUs.

### 4.6. Crypto Tool

To make more accurate performance measurements, we created an implementation of NAC, named Crypto Tool. This tool was used in the evaluation performance of hash functions and signature operations, the details of which are presented below.

Our protocol requires a hash function to generate implicit messages. To choose the best option for our implementation, we did a performance test with well known hash functions, intensively used by the cryptographic community [41,42,43,44]. In Table 1 and Table 2 are the performances of hash functions obtained in an AMD Opteron Processor 4238 3.3 GHz processor with two different implementations (Crypto++ and OpenSSL).

The analysis of the efficiency performance over hash functions points out that MD5 is the most efficient hash function. Nonetheless, efficiency is not the only evaluation metric. Researchers have proved that it is possible to perform second pre-image attacks to MD5’s hashes [43]. NAC does not require second pre-image resistance properties in a hash function. In fact, the only required property by NAC is pre-image resistance. It is important that upon receiving an implicit message $H\left(i\right)$ an attacker is not capable of calculating *i*. Moreover, the second pre-image attacks are useless against NAC. Let us assume an attacker exploits *H* and discovers an i2 such that i≠i2 and $H\left(i\right)=H\left(i2\right)$. Upon broadcasting i2, the attacker would only be confirming the message disclosed by the RSU.

Furthermore, these attacks can not be executed within the timing constraints that NAC imposes. In order to deliver accurate information to the OBUs, the validity interval must be in the order of magnitude of 10 s at most. The second pre-image attacks take more than that, and they also require significant computational power. For these reasons, we choose to use MD5 as the cryptographic hash function used to generate implicit messages.

To choose a signature algorithm for anchor messages, we evaluated five alternatives implemented by Crypto++, the results of which are in Table 3. It is possible to see that only RSA 1024 executes faster than ECDSA B-233 and ECDSA P-256. However, it is also known that the usage of RSA 1024 is not recommended.

After Snowden’s revelations in 2013, some doubts emerged about NIST’s curves (https://apps.nsa.gov/iaarchive/programs/iad-initiatives/cnsa-suite.cfm). These revelations raised suspicions that NSA had intentionally added back-doors on a pseudo-random number generator. Those suspicions led many corporations not to use NIST’s curves. For the above reasons, our implementation of the NAC protocol uses Curve25519/Ed25519 [45] to handle digital signatures on anchor messages.

A crucial aspect of the validation of anchor messages’ signatures is the pre-knowledge of RSUs’ public keys by OBUs and the trust on the correctness of those keys. In this paper, we assume that both knowledge and trust are ensured by some form of secure pre-loading, but we do not detail how that could be achieved.

## 5. Performance Evaluation

We started the NAC evaluation by message size, validation times and radio environment occupancy; three important metrics. They show the impact on the network, the time requirements for notifying drivers and the system scalability. After that, we discuss some performance aspects of NAC in the real scenario based on Aveiro City, using patterns of displacement of tourists in this Portuguese city. We ended by demonstrating how NAC can be used as a city management tool discussing the better distribution of RSUs in the city’s tourist circuit.

### 5.1. Message Size

**Figure 8:**
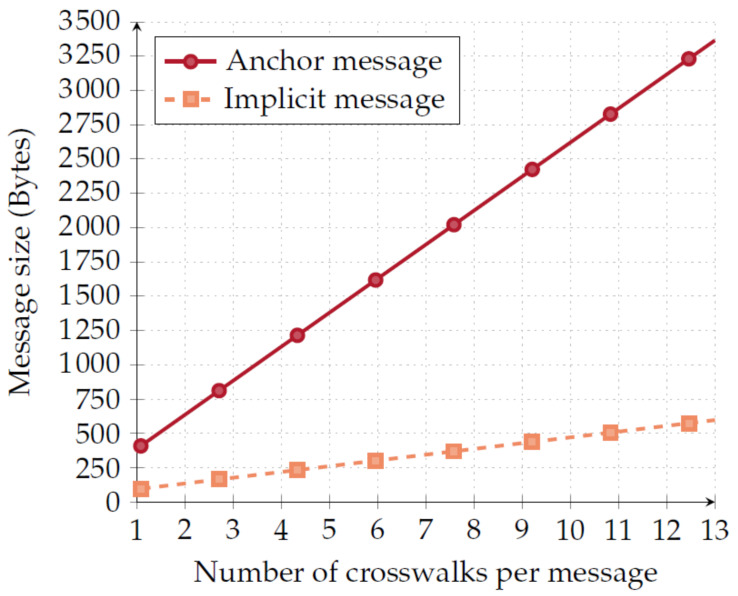
Message size x number of crosswalks.

Message size is an important metric of our system’s evaluation, as it can increase network overhead and affect dissemination time. That way, we start by assessing the impact of message size. After performing our first tests, we concluded that the number of crosswalks associated with an RSU is linear with respect to the size of both the anchor message and the implicit message. In Figure 8, we depict the size of the two types of messages.

It is important to notice that anchor messages are considerably larger than implicit messages given the fact that they carry a lot more information than implicit messages. Most of this information is either

(i) Static information, such as geo-location of both the crosswalks and the RSU; and
(ii) Timing information, including all of the times that are required for the OBU to validate implicit messages.

Moreover, anchor messages are asymmetrically signed. The signature’s size alone is 88 bytes long, after *base64* encoding implicit messages only carry information regarding the current state of the crosswalk. This allows implicit messages to be significantly smaller.

### 5.2. Validation Times

Due to vehicle mobility, any delay in message delivery may affect the effectiveness of the system, as vehicles may not receive it at a distance from which they may be able to react. This makes validation time an important metric for our system. Thus, one of our main goals was to develop a system that does not require a lot of computational time to authenticate and interpret the occupation status of the crosswalk. We use our Crypto tool (Section 4.6) to understand the evolution of validation time while increasing the number of crosswalks. The points of the Figure are an average of 10,000 runs.

**Figure 9:**
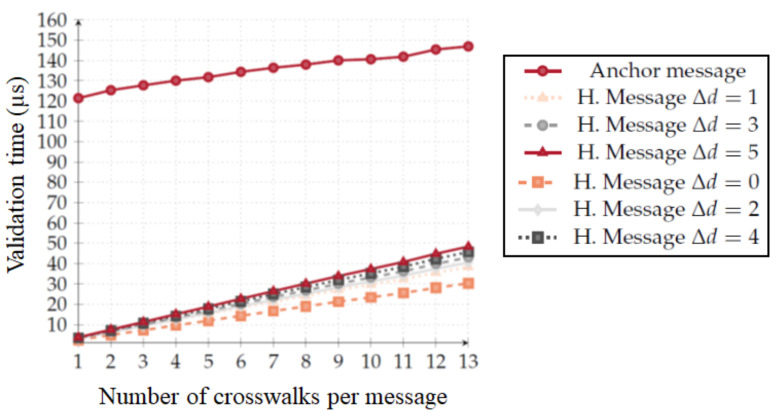
Evolution of validation time x number of crosswalks.

Figure 9 depicts the relationship between the number of crosswalks and the message validation time. For implicit messages, we consider several interval scenarios (Δd). For example, Δd=0 occurs if an OBU receives an implicit message that it had already received, and Δd=3 occurs when the current time interval is three time intervals ahead of the last received hash chain message. Firstly, this graph depicts that implicit message authentication is considerably faster when compared with anchor message authentication. In the most critical scenario, assuming that the OBU is five validity intervals behind (Δd=5) and that the RSU is disseminating information about the thirteen crosswalks, implicit message validation is 3.04 times faster then anchor message validation.

**Figure 10:**
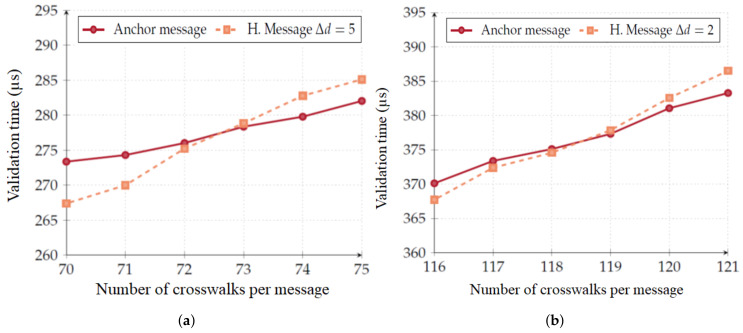
Comparison of anchor message validation against implicit message validation. (**a**) 73 crosswalks and five validity intervals. (**b**) 119 crosswalks and two validity intervals.

It is important to note that anchor message validation is only dependent on the message size and, consequently, it is only dependent on the number of crosswalks. Implicit message validation, however, is performed on a per crosswalk basis. Therefore, if the number of crosswalks an RSU is responsible for is high enough, implicit message validation may become heavier to perform when compared to anchor message validation. Nevertheless, this situation would only occur if an RSU was responsible for:

- 73 crosswalks if the OBU is five validity intervals behind (Figure 10a); and
- 119 crosswalks if the OBU is two validity intervals behind (Figure 10b).

By observation of the real situations and considering works such as [46,47], we consider that an RSU can be responsible for up to 13 crosswalks in a 100 m radius. We understand that this perimeter is appropriate for the effective dissemination of warning messages.

### 5.3. Mobility Scenario

To better understand the aspects related with radio environment occupancy, it is necessary to discuss some details about our mobility scenario.

**Figure 11:**
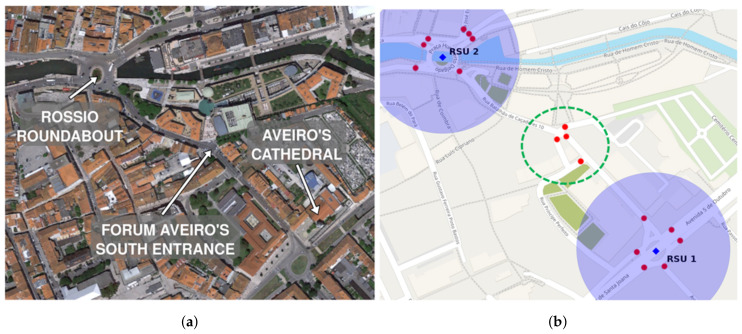
Mobility scenario. (**a**) Aveiro city, Portugal. (**b**) Used crosswalks (red marks).

In Figure 11a, we show the part of Aveiro that we used to test our scenario along with labels of the most significant places. Aveiro is in the process of becoming a smart city (https://uia-initiative.eu/en/uia-cities/aveiro), and these test elements can be crucial to its development.

This area is interesting due to the number of crosswalks it possesses. It is a very popular area among tourists. In Figure 11b, we depict the existent real crosswalks that we used in our test scenario.

### 5.4. Radio Environment Occupancy

WAVE technology is currently the most widely used in V2X communications within the scope of vehicular networks. It is very important to take into consideration its radio environment occupancy mainly because low radio channel occupancy levels allow other communication systems to reach a better performance ratio, and a high radio channel occupancy may implicate the loss of messages.

As described earlier, one of the measures we took to reduce radio pollution is the reduced signal power usage. RSUs can disseminate with higher signal powers. However, high signal power levels contribute to higher radio environment occupancy. Moreover, in our scenario, if an OBU receives a message when it is located 100 m from the crosswalk, it can stop the vehicle in time. This is in agreement with distances used in the literature, such as 40 m for speed breaker, 52 m in school zones, and others [48].

In Section 5.5.1, we discuss in detail the correlation of NAC message validation and the mean validation distance. Now we will make a theoretical approach in order to test the radio environment usage varying the amount of existing RSUs. This will be done through two scenarios:

(i) Each crosswalk has an RSU that is responsible for message dissemination, taking into account the current state of that crosswalk; and
(ii) There are only two RSUs responsible for all the crosswalks in the region of interest. RSUs are blue squares in the center of the shadow circles shown in Figure 11b.

The number of crosswalks has a clear impact on message size, which, consequently, has an impact on radio environment usage. Nevertheless, we will leave this fact aside for now. For simulation purposes, we evaluated two options in our scenarios:

(i) Each RSU sends *five* messages per second; and
(ii) Each RSU sends *twenty* messages per second.

**Figure 12:**
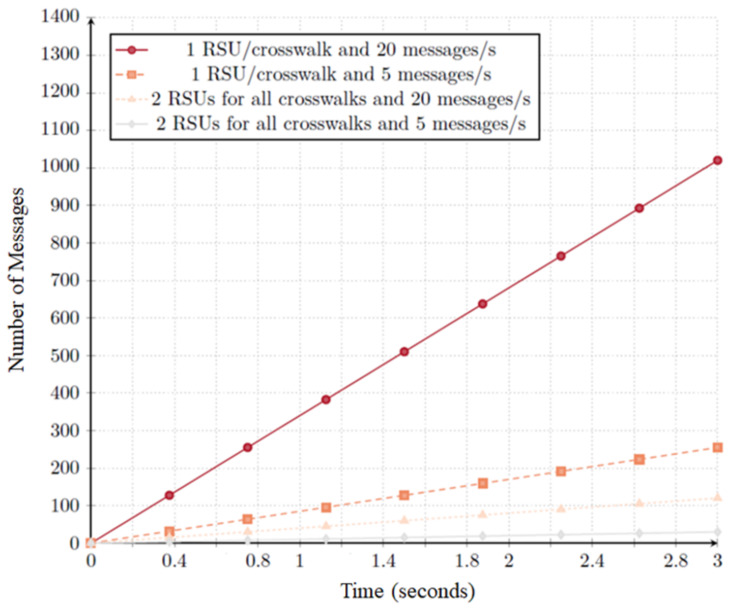
Number of messages sent by Road Side Units (RSU) per second.

The depicted graph of Figure 12 shows that an increasing number of RSUs has significant impact on the number of disseminated messages/s. A considerable number of RSUs not only increases the number of messages/s, but also increases the number of irrelevant messages per second if we consider that a single RSU is capable of providing the same information in the same time. Moreover, message dissemination intervals have a key influence in the delivery of crosswalk state changes. Therefore message dissemination intervals are important factors that we will discuss in detail later on.

By implementing an RSU per crosswalk, the number of messages increases significantly given that each RSU will disseminate the current state of its crosswalk. Considering 1 RSU per crosswalk for 17 crosswalks, we would have *f(x)* = (17 × 20)*x* with 20 messages per second, and *f(x)* = (17 × 5)*x* for 5 messages per second. Considering the above, we conclude that RSU placement is critical to inform the network nodes about crosswalk state. A proper RSU placement allows for messages to be delivered with considerable anticipation in order to inform vehicles about crosswalks ahead. Moreover, the number of crosswalks an RSU is responsible for influences the size of the disclosed messages. Message size also influences radio environment usage. Nonetheless, its impact is not as considerable as the impact of different messages (mostly because of environment access controls implemented by the WAVE protocol).

### 5.5. Scenario Evaluation

Following, we will discuss in detail the protocol’s impact on a mobility scenario. In Figure 11b, we depict the positioning of both crosswalks (red dots inside purple circles) and RSUs (blue squares).

The location of the RSU 2 is the main tourist and commercial point of Aveiro. There is the main shopping (Forum Aveiro) and the old town (Rossio). The RSU 1 is also close to two points of interest (Aveiro Museum and Cathedral) and is close to a student’s zone in which mobility is characterized by specific peak periods typical of the mobility of local citizens. We use this scenario to evaluate the interval between message disclosure and the anchor message dissemination frequency.

We start by clarify the impact of *Validity* and *Message Disclosure* intervals over the system performance. We considered the two combinations shown in Table 4. The first scenario uses a validity interval of 200 ms and discloses messages every 50 ms. The second scenario uses validity intervals of 1000 ms and discloses messages every 200 ms. The first test runs for 30 s total and the second test runs for 60 s total. As a simplification, we may refer to the term *validity interval* as *d* later on.

The second test aims to elucidate if the *variation of anchor message update* has any impact on the system. To test the impact of anchor message dissemination, we defined three different frequencies, sending one anchor message for every 15 messages, for every 30 messages, and for every 40 messages. As a simplification, we may later on refer to the term *anchor Message disclosure interval* as ***adi***.

The variation of both validity intervals and anchor message frequency should have real impacts on the system. By increasing the size of validity intervals, we force the RSU to broadcast the same message for longer intervals. Even if the state of the crosswalks changes in the meantime, if the RSU already broadcast a message stating the presence (or not) of pedestrians in the crosswalk, it is forced to maintain that same state until the validity interval ends. Therefore, validity intervals should be small enough to enable drivers to receive the state of the crosswalk closer to real-time. Moreover, the update of anchor messages also has a real impact on the system performance. New vehicles in the network must receive an anchor message update in order to be able to validate implicit messages. Therefore, anchor messages disclosure is a trade-off between vehicle information and heavier message validation.

#### 5.5.1. Mean Vehicle Warning Distance

Using the features of the simulation platform, we want to estimate how far from the crosswalk vehicles receive the valid alert message indicating the presence of pedestrians.

**Figure 13:**
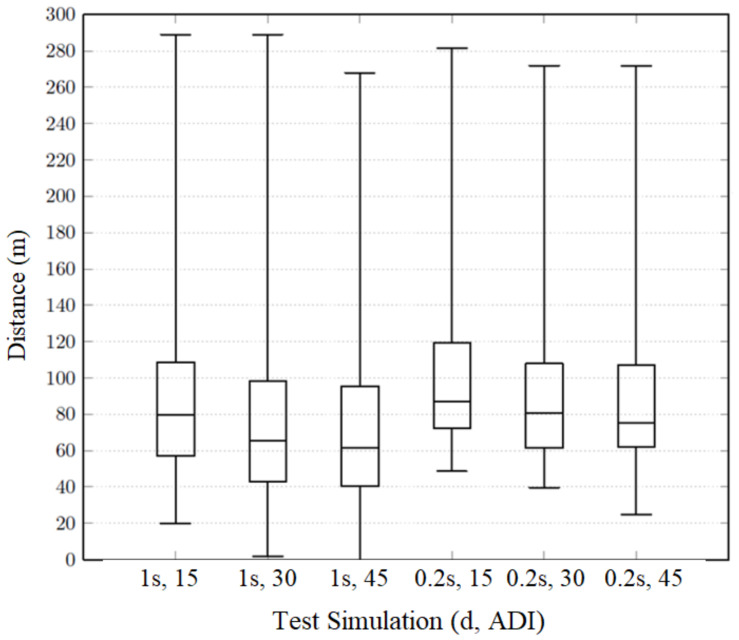
Warning distance of the vehicles.

For each test performed, Figure 13 depicts five horizontal lines:

1. The middle line is the median of the values, also known as second quartile ($Q_{2}$);
2. The upper and lower bounds are the maximum value and the minimum value, respectively;
3. The first quartile ($Q_{1}$) is defined as the middle number between the smallest number and the median; and
4. The third quartile ($Q_{3}$) is the middle value between the median and the highest value.

As predicted by theoretical analysis, by sending anchor messages with higher frequency, the OBUs are informed about the two hash chains earlier and, therefore, can authenticate messages with anticipation. Smaller validity intervals (and, consequently, higher message disclosure frequencies) allow OBUs to be warned about crosswalk states earlier. The median is, on average, 23% bigger on 200 ms tests than on 1 s tests. It is possible to conclude that OBUs get warned closer to the crosswalks when the system uses

(i) Smaller validity intervals, and, consequently,
(ii) Smaller message disclosure intervals, and
(iii) Smaller anchor update intervals.

It is important to point out that Figure 13, does not consider OBUs that only receive information after passing the crosswalk. Vehicles warned about crosswalks’ existence after passing the crosswalk may return a small distance. If these distances were considered, the graph would present unreliable results. For this reason, the graph only considers vehicles that are warned before passing through the crosswalk. With this in mind, we considered it pertinent to analyze the percentage of crosswalks getting validated after passing through the crosswalk, therefore receiving unhelpful information and not having information regarding that specific crosswalk with due anticipation. The results for validity intervals of 200 ms and 1000 ms are depicted in Table 5a.

Although the percentage of crosswalks validated before vehicles pass through them is very high, there is still some percentage of crosswalks that are validated only after the OBU passes through it (an average of 6.3% of the crosswalks). After testing the system, we concluded that the four crosswalks located in the middle of the map (dashed green circle in Figure 11b) were causing this issue. The problem relies on the fact that they are considerably distant from **RSU 1**, which is the RSU that CAT assigns to be responsible for them. This way, when vehicles are approaching these crosswalks, in the direction from **RSU 2** to **RSU 1**, they do not receive the message in a relevant time. In Table 5b, we depict the percentage of crosswalks validated before vehicles pass through them, this time considering only those four crosswalks.

The depicted percentages drop considerably when isolating the four crosswalks located in the middle of the map with no dedicated RSU. It is possible to conclude that these crosswalks contribute significantly to the rate of messages received late. Moreover, notice that the four crosswalks are placed 200 m away from the RSU; therefore, this does not represent a system problem but a scenario obstacle. To better understand the particular characteristics of our scenario, we developed a scenario including an extra RSU for those four crosswalks exclusively. That scenario is depicted in Section 5.6.

### 5.6. Management Distribution of Resources Across the City

**Figure 14:**
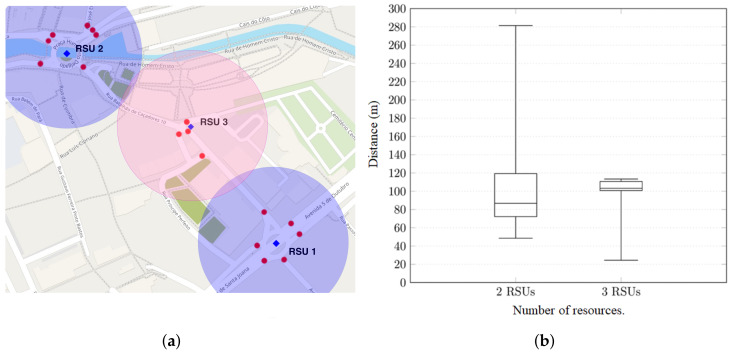
Enhanced scenario with 3 RSUs and their evaluation compared with the previous scenario. (**a**) Scenario with the third RSU. (**b**) Comparison between scenarios (2 × 3 RSUs).

As shown earlier, NAC can help to verify the distribution of RSUs throughout the city. Using its metrics, it was possible to identify a coverage gap in a group of crosswalks. Our intuition tells us that a new RSU inserted nearby four isolated crosswalks can solve the potential risk. Taking this into account, we developed a new scenario that introduces an RSU near the south entrance of Forum Aveiro, halfway between the two RSUs in the previous scenario. That scenario is depicted in Figure 14a.

Figure 14b depicts the comparison of the best results obtained in Figure 13. In the tests with the improvement scenario, we use *d* = 200 ms and *adi* = 15 messages.

The emulation of the new scenario shows better results:

- The median value is 3% higher when using the new scenario;
- The maximum warning distance is substantially reduced (approximately 0.46 smaller) when using the third RSU. This way vehicles that are outside of the crosswalk range are not unnecessarily warned about the existence of crosswalks they may not pass through; and
- The percentage of warned vehicles also increased. With the inclusion of a new RSU, 100% of the existent vehicles were warned before passing through any crosswalk.

These results show, once again, that RSU positioning relative to the crosswalks and to the vehicles is of high relevance for a good system performance. The NAC can assist the management of the city in the search for the best distribution of its resources.

### 5.7. Message Loss Scenario

The mobility and connectivity tests performed in the above sections do not account for message loss scenarios. In fact, the earlier experiments assume messages are sent without transmission errors. In order to test for more realistic situations, we decided to deliberately introduce a message loss percentage.

In the following tests, we used a validity interval of 200 ms, a message dissemination time of 50ms, and the scenario depicted in Figure 14a using three RSUs.

**Figure 15:**
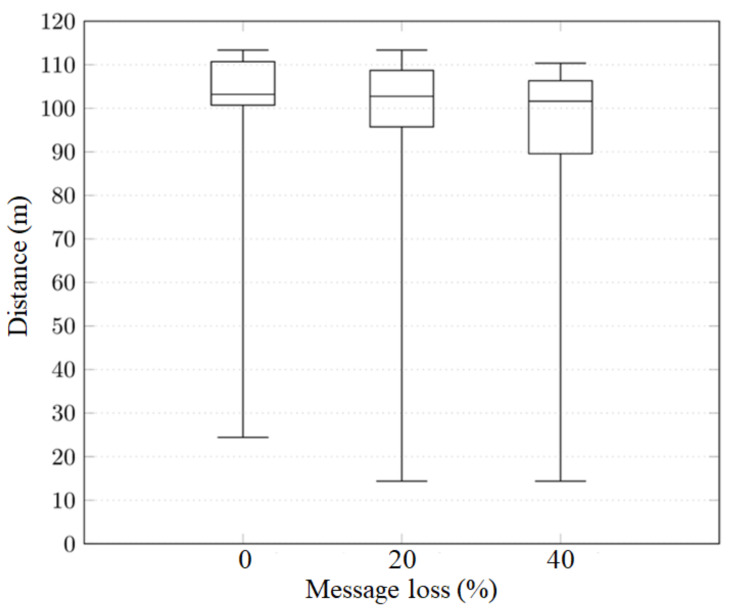
Message loss evaluation.

As expected, the validation distance of the vehicles dropped, as shown in Figure 15. The maximum validation distance remained constant at 113.36 m. The minimum distance dropped from 24.43 m at 0% message loss, to 14.37 m at 20% message loss and then was maintained at 14.37. However, the median only dropped around 1% per 20% lost messages increase. In fact, anchor message loss has an impact on the distance a vehicle is warned. The bigger the message loss probability, the smaller the warning distance is.

## 6. Conclusions and Future Work

As in any other innovation process, smart cities require the development of dedicated solutions that focus on their particular requirements. To contribute to this process, we presented the design and exploitation of Nimble Asymmetric Cryptography (NAC), a protocol that was designed to broadcast secure information messages containing a limited set of information status. This protocol was used to inform drivers about the presence of pedestrians in nearby crosswalks.

NAC was specifically designed to meet the requirements of urban communication infrastructures in smart cities, where VANETs are becoming progressively interesting. NAC presents a reliable alternative addressing the main characteristics of VANETs’ communication, such as mobility, intermittent contact between nodes, and changes in topology. Moreover, while accounting for VANET characteristics, NAC also cares for the strong security requirements, such as an efficient, non-repudiation mechanism for transmitted messages.

As our results showed, NAC offers high-speed cryptographic security, even when considering devices with low computing power, which is appropriate for IoT environments. Furthermore, the variation of message dissemination times and validity intervals presented comply with the IEEE 802.11p standard for wireless communication, where each vehicle disseminates a message within a time interval (100–300 ms) to other vehicles or to RSUs.

Through the experiments done in our realistic scenario, where we used crosswalks of the city of Aveiro, Portugal, we were able to evaluate the efficiency in the distribution of the messages. Using NAC, it was possible to identify that, in order to increase NACs performance, a proper RSU placement is critical, involving both the crosswalks and the vehicles in the RSU radius.

Concluding, NAC takes advantage of time constraints and the distinctiveness of hash chains in order to achieve an efficient authentication of broadcast messages, while maintaining message loss tolerance. This set of advantages can be of much use in smart cities, where many sensors broadcast a finite set of values, and in scenarios where message validation times can make the difference between life or death.

As future work, we intend to expand the applications of NAC in the IoT universe. There are a variety of sensors in smart cities that follow a similar principle (traffic lights, environmental sensors, trash sensors, etc.), and thus are suitable for being handled using NAC. As such, we aim to evaluate the requirements for easy customization of the NAC to make it a popular solution.

## Figures and Tables

**Table 1:** Crypto++ self-assessment of its hash functions’ performance.

Algorithm	MiB/Second	Cycles/Byte
**MD5**	74	42.3
**SHA-1**	29	108.1
**SHA-256**	20	154.6
**SHA-512**	24	132.9

**Table 2:** OpenSSL self-assessment of its hash functions’ performance.

	KB/Second per Input Size				
Algorithm	16 B	64 B	265 B	1 KiB	8 KiB
**MD5**	7748.71	21,039.56	154,707.68	488,783.03	559,373.40
**SHA-1**	7432.60	19,319.64	264,589.06	475,826.60	663,834.20
**SHA-256**	4489.59	9681.25	16,430.99	19,764.60	21,067.14
**SHA-512**	3646.80	14,791.76	21,917.44	30,640.82	34,465.11

**Table 3:** Crypto++ signature performance, for both creation and verification, on an AMD Opteron Processor 4238 3.3 GHz processor.

	Creation		Verification	
Algorithm	Milliseconds	Megacycles	Milliseconds	Megacycles
**RSA 1024**	0.473	1.561	0.069	0.228
**RSA 2048**	16.129	53.226	0.406	1.338
**ed25519**	0.195	0.645	0.161	0.532
**ECDSA B-233**	13.807	45.564	17.241	56.897
**ECDSA P-256**	0.907	2.994	3.074	10.143

**Table 4:** Time parameters used to perform test results.

Validity Interval	Message Disclosure Interval	Total Simulation Time
(ms)	(ms)	(s)
200	50	30 s
1000	200	60 s

**Table 5:** Validated message percentage.

a Crosswalks validated on time (%).		
**Anchor Dissemination Interval (msgs)**	**Valid Message (%)**	
	**200 ms**	**1000 ms**
15	0.97	0.94
30	0.97	0.92
45	0.96	0.86
**b Four crosswalks without RSU cover.**		
**Anchor Dissemination Interval (msgs)**	**Valid Message (%)**	
	**200 ms**	**1000 ms**
15	0.71	0.65
30	0.71	0.65
45	0.62	0.64
